# Exploration of the Sphingolipid Metabolite, Sphingosine-1-phosphate and Sphingosine, as Novel Biomarkers for Aspirin-exacerbated Respiratory Disease

**DOI:** 10.1038/srep36599

**Published:** 2016-11-10

**Authors:** Hoang Kim Tu Trinh, Su-Chin Kim, Kumsun Cho, Su-Jung Kim, Ga-Young Ban, Hyun-Ju Yoo, Joo-Youn Cho, Hae-Sim Park, Seung-Hyun Kim

**Affiliations:** 1 Department of Allergy and Clinical Immunology, Ajou University School of Medicine, Suwon, Korea; 2 Translational Research Laboratory for Inflammatory Disease, Clinical Trial Center, Ajou University Medical Center, Suwon, South Korea; 3 Department of Pharmacology and Therapeutics, Seoul National University College of Medicine and Hospital, Seoul, Korea; 4 Asan Institute for Life Sciences, Asan Medical Center, University of Ulsan College of Medicine, Seoul, Korea

## Abstract

Sphingolipid (SL) metabolites have been suggested to be important inflammatory mediators in airway inflammation and asthma. However, little is known about SL metabolites in aspirin-exacerbated respiratory disease (AERD). We aimed to explore the potential AERD biomarkers by conducting lipidomics targeting SL metabolites. The levels of SL metabolites in serum and urine samples from 45 AERD patients and 45 aspirin-tolerant asthma (ATA) patients were quantified through mass spectrometry. During the lysine-aspirin bronchoprovocation test (ASA-BPT), the levels of serum sphingomyelin (SM) were significantly decreased in AERD (*P* < 0.05) but not in ATA. The serum SM levels were positively correlated with airway responsiveness to methacholine. At the basal status before the ASA-BPT, the levels of serum sphingosine-1-phosphate (S1P) and urine sphingosine were significantly higher in the AERD patients compared with that of ATA patients (*P* < 0.001) and were positively correlated with a greater decrease in FEV_1_ (%) values following the ASA-BPT test (*P* < 0.001 for each), and with serum periostin level (*P* < 0.05 for each). This study is the first to evaluate serum S1P and urine sphingosine as potential biomarkers of AERD as well as to examine the metabolic disturbance of SL in AERD patients.

Sphingolipids (SLs) are ubiquitous cell membrane components that regulate multiple cellular processes, such as cell division, differentiation, apoptosis and autophagy^1^,^2^. The metabolism of SL is a complex pathway that generates sphingosine-1-phosphate (S1P) from various precursors, using ceramide as a backbone. Ceramide is produced from either *de novo* SL synthesis or sphingomyelin (SM)^3^. Ceramide is deacetylated by ceramidases to produce sphingosine, which is then phosphorylated by sphingosine kinase types 1 and 2, producing S1P^3^.

The potential roles of SL metabolites in respiratory diseases have been discussed, although there is some debate^4^,^5^. Acid sphingomyelinase, an enzyme of SM hydrolysis, is involved in endothelial dysfunction, cytokine responses and neutrophils sequestration during acute lung injury^5^. Decreased serine palmitoyl-CoA transferase (SPT) activity is associated with methacholine-induced airway hyperreactivity^6^. Ceramide expression has been detected in patients with emphysema and chronic-obstructive pulmonary disease^7^.

Previous studies linking asthma to SL have focused on airway inflammation and allergic responses related to S1P. S1P can initiate mast cell activation and chemotaxis as well as amplify mast cell degranulation by interacting with S1P receptors, primarily S1P_1_ and S1P_2_^8^,^9^. An *in vivo* study has reported that S1P treatment induced secretion of T-helper 2 lymphocyte (Th2) and Th17 cytokines, such as interleukin-4 (IL-4) and IL-17, respectively^10^. S1P has also been associated with methacholine-induced airway hyperreactivity, bronchoconstriction and airway remodeling by targeting genes regulating cell proliferation^4^,^11^,^12^.

Asthma is a common and clinically heterogeneous disorder that is characterized by intermittent airflow obstruction, airway hyperreactivity, chronic airway inflammation, and increased mucus production^13^. Because of its heterogeneity, asthma is hard to explain by a single mechanism. Lotvall *et al*. have introduced the definition “endotype” to define different asthma subtypes, based on their mechanisms^14^. Asthma could be classified into six endotypes (depending on the disease characteristics), including aspirin-exacerbated respiratory disease (AERD) and aspirin-tolerant asthma (ATA). AERD, also termed “aspirin sensitive asthma”, is a syndrome triggered by ingesting non-steroidal anti-inflammatory drugs (NSAIDs) that is associated with an increased production of cysteinyl leukotrienes (CysLTs). AERD is likely to be a more severe case of asthma, with a higher frequency of emergency visits/hospitalizations and increased mortality versus allergic asthma^15^,^16^,^17^. However, we still lack good biomarkers to differentiate AERD from ATA.

Given that SLs, particularly S1P, could contribute to the pathogenesis of airway inflammation and asthma, we aimed to investigate the SL profiles of AERD patients and its association with clinical outcomes. The serum and urine samples from AERD and ATA patients were analyzed for SL metabolites using lipid chromatograpy- mass spectrometry. Additionally, we searched for a correlation between SL metabolites and key AERD biomarkers, 15-hydroxyeicosatetraenoic acid (15-HETE)^18^, human leukocyte antigen HLA-DBP1*0301^19^, cysteinyl leukotriene receptor type 1 (*CYSLTR1)* gene polymorphism^20^ and periostin^21^. To discriminate AERD from ATA, receiver operating characteristics (ROC) curve analysis for serum S1P and urine sphingosine were analyzed.

## Results

### Patient characteristics

Table 1 shows the clinical characteristics of the study subjects. The AERD patients showed a greater decrease in FEV_1_ (%) following the Lysine-Aspirin bronchoprovocation test (ASA-BPT) than the ATA patients (*P* < 0.001). The AERD patients were younger than the ATA patients (*P* = 0.045). The AERD patients showed a larger decline in FEV_1_ (%) predicted values following the ASA-BPT and a lower PC_20_ value than the ATA patients (*P* < 0.001 and *P* = 0.004, respectively). The AERD patients had higher frequencies of rhinosinusitis and nasal polyposis compared with the ATA patients (86.7% vs. 61.9%, *P* = 0.013 and 37.8% vs 16.7%, *P* = 0.033, respectively). There were no significant differences between the AERD and ATA patients in terms of the other baseline characteristics.

### Correlation of the serum SM levels with methacholine-induced airway hyperreactivity

The serum and urine SL compositions for the AERD and ATA patients during the ASA-BPT are provided in Table 2. To determine how SL metabolites changed following the ASA-BPT, we used star and symbol plotting to consider the induction fold of SL metabolites following the ASA-BPT in the AERD and ATA groups (Fig. E1). The SL profiles of the AERD group showed distinct patterns compared with those of the ATA group. Distorted increased patterns of ceramides and SMs after the ASA-BPT were characteristic for the ATA group, while decreased patterns of ceramides and SMs were observed in the AERD group following the ASA-BPT.

**Figure 1:**
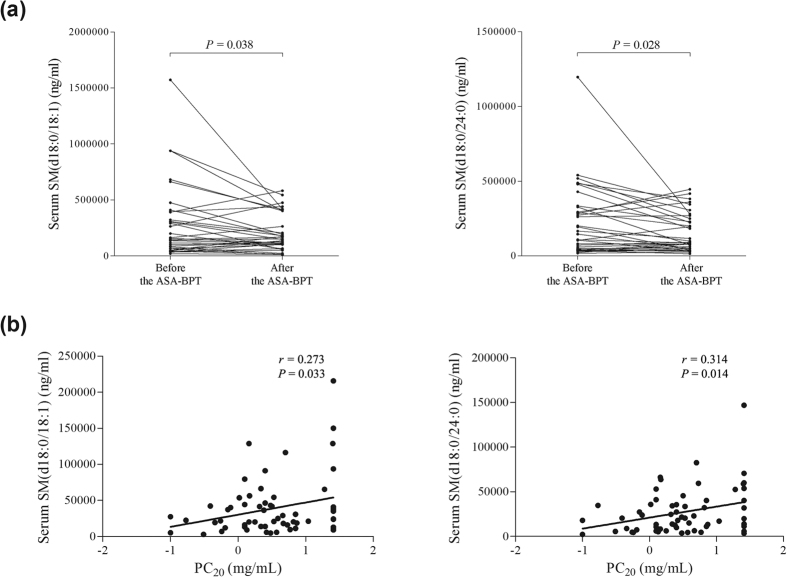
Changes in serum Sphingomyelin (SM) levels during the ASA-BPT in correlations with methacholine- induced airway hyperreactivity. (**a**) Change in serum SM level following the ASA-BPT in the AERD group, (**b**) positive correlations of the serum SM levels at the basal status with PC_20_ to methacholine. Log-transformed data of PC_20_ to methacholine was analyzed.

Comparing changes in the SL metabolite levels during the ASA-BPT, the levels of serum SMs (d18:0/18:0, d18:0/18:1, d18:0/24:0, d18:0/24:1) were significantly decreased during the follow-up after exposure to ASA through the ASA-BPT in the AERD group (*P* < 0.05 for each, Fig. 1a, Table 2), but not in the ATA group (*P* > 0.05 for each, Table 2). The levels of serum SMs (d18:0/16:0, d18:0/18:1, d18:0/24:0, Lyso SM) showed positive correlations with methacholine-induced airway hyperreactivity (*P* = 0.051, *P* = 0.067, *P* = 0.033, *P* = 0.014, *P* = 0.071, *P* = 0.050 for each, Fig. 1b, Table 3). Regarding S1P, the levels in serum and urine samples were increased significantly following the ASA-BPT in both AERD and ATA groups (*P* < 0.05 for each, Table 2).

### Serum S1P and urine sphingosine as potential biomarkers to differentiate AERD from ATA

**Figure 2:**
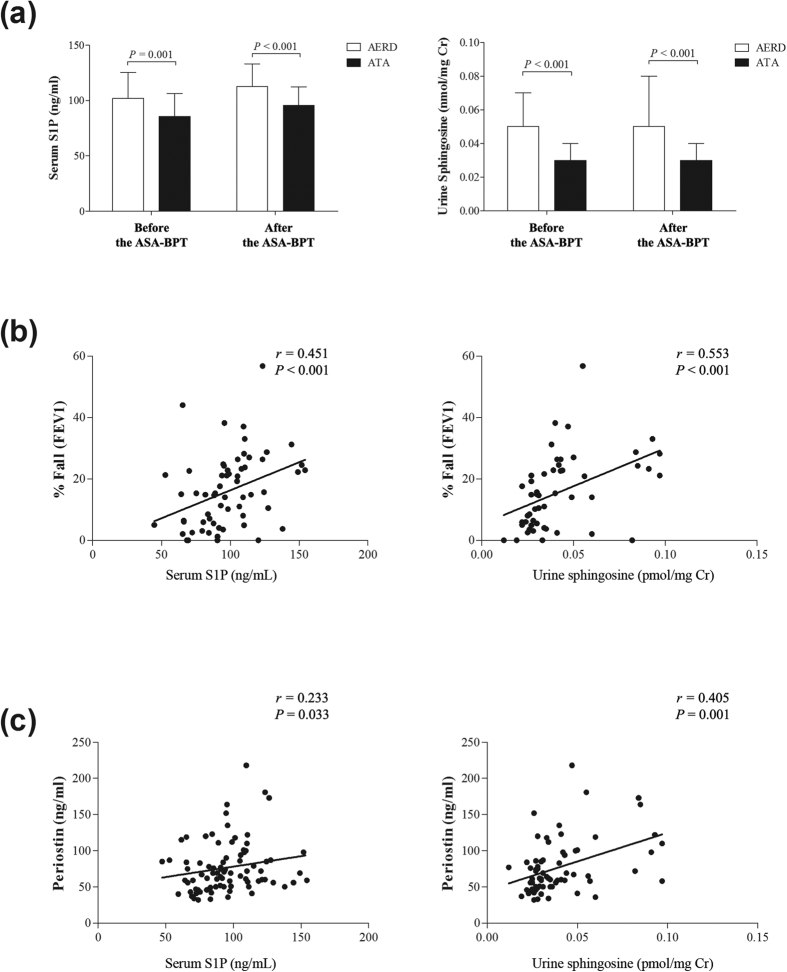
Comparison of the serum S1P and urine sphingosine levels (**a**) between the AERD and ATA groups, data are presented as means ± SD. Correlation of the basal status of serum S1P and urine sphingosine levels with (**b**) the decline of FEV_1_ (%) following the ASA-BPT, and (**c**) serum periostin level.

The serum S1P and urine sphingosine levels were significantly higher in the AERD group than in the ATA group at the basal status, before the ASA-BPT, and at onset of reaction symptoms after the ASA, following the ASA-BPT (*P* < 0.001 for each, Fig. 2A).

To assess the relationship between the SL metabolites and airway inflammation status, the correlation between the SL metabolites levels at the basal status before the ASA-BPT test and baseline clinical characteristics and biomarkers in asthmatic patients were evaluated (Table 3). The levels of serum S1P and urine sphingosine at the basal status before the ASA-BPT test were significantly correlated with a greater decrease in FEV_1_ (%) values during the ASA-BPT (*r* = 0.451, *P* < 0.001; *r* = 0.553, *P* < 0.001, respectively, Fig. 2B).

We investigated the correlation with serum periostin, which was known as a novel biomarker of AERD. At the basal status before the ASA-BPT test, the urine sphingosine level was positively correlated with the serum periostin level, however, the serum S1P showed a weak correlation with the basal status of serum periostin (*r* = 0.405, *P* = 0.001; *r* = 0.233, *P* = 0.033 respectively, Fig. 2C).

**Figure 3:**
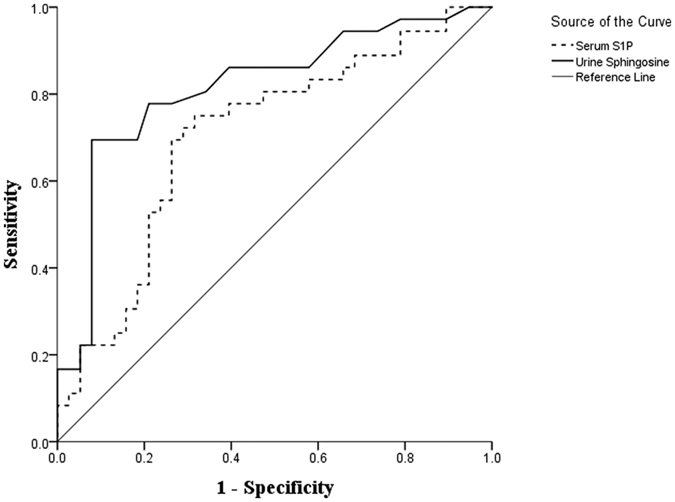
ROC analysis for the basal status of serum S1P and urine sphingosine levels to predict AERD. The continuous line represents urine sphingosine and the dashed line represents serum S1P.

Using the ROC method, we evaluated the values of the serum S1P and urine sphingosine levels as diagnostic biomarkers for AERD (Fig. 3). To generate the highest levels of sensitivity and specificity, the optimal cut-off values from the ROC curve, a serum S1P > 93.5 ng/mL and urine sphingosine 33.5 pmol/mg Creatinine (pmol/mg Cr) was chosen. When these values were set, the serum S1P showed 72.2% sensitivity/71.1% specificity (AUC = 0.705, *P* = 0.002) and urine sphingosine showed 77.8% sensitivity/78.9% specificity, (AUC = 0.816, *P* < 0.001) for discriminating AERD from ATA.

We also examined the correlation between the SL metabolites at the basal status and 15-HETE which has been known to be as a diagnostic biomarker for AERD. The serum 15-HETE level was positively correlated with serum sphingosine (*r* = 0.459, *P* < 0.001, Table 3), while negatively correlated with serum SMs (d18:0/16:0, d18:0/18:0, d18:0/18:1, Lyso SM) (*r* = −0.338, *P* = 0.005; *r* = −0.299, *P* = 0.013, *r* = −0.263, *P* = 0.03; *r* = −0.282, *P* = 0.020 respectively, Table 3).

### Correlation between the SL metabolites

**Figure 4:**
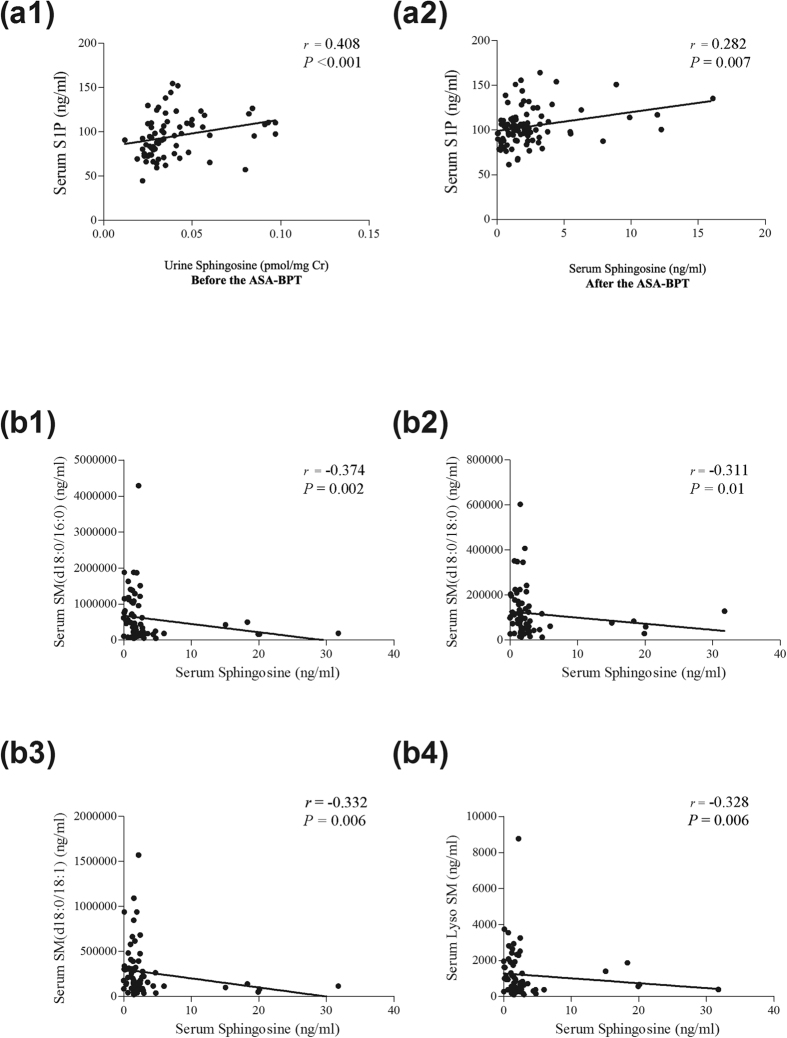
Correlation between the SL metabolite levels. (**a**) Positive correlation of (a1) serum S1P and urine sphingosine at the basal status before the ASA-BPT, (a2) serum S1P and serum sphingosine at onset of reaction symptoms following the ASA-BPT. (**b**) Negative correlation of serum sphingosine level at the basal status with (b1) serum SM (d18:0/16:0), (b2) with serum SM (d18:0/18), (b3) with serum SM (d18:0/18:1), (b4) with serum LysoSM before the ASA-BPT.

Next, we assessed the correlation between the SL metabolites. The correlation coefficients between the SL metabolites are provided in Supplementary Tables E1 and E2. At the basal status before the ASA-BPT test, the serum S1P level was positively correlated with urine sphingosine level (*r* = 0.408, *P* < 0.001, Fig. 4, a1). After the onset of reaction symptoms following ASA-BPT, serum S1P level was correlated weakly with serum sphingosine level (*r* = 0.282, *P* = 0.007, Fig. 4, a2). The level of serum sphingosine at the basal status was negatively correlated with the level of serum SMs (d18:0/16:0, d18:0/18:0, d18:0/18:1, Lyso SM) (Fig. 4, b1–4, Table E1). The serum SM levels were positively correlated with the levels of ceramides (Table E1). The urine SM levels were positively correlated with the levels of urine ceramides (Table E2).

### Association of the urine sphingosine level with HLA-DBP1*0301 and CYSLTR1-634C > T polymorphism

**Figure 5:**
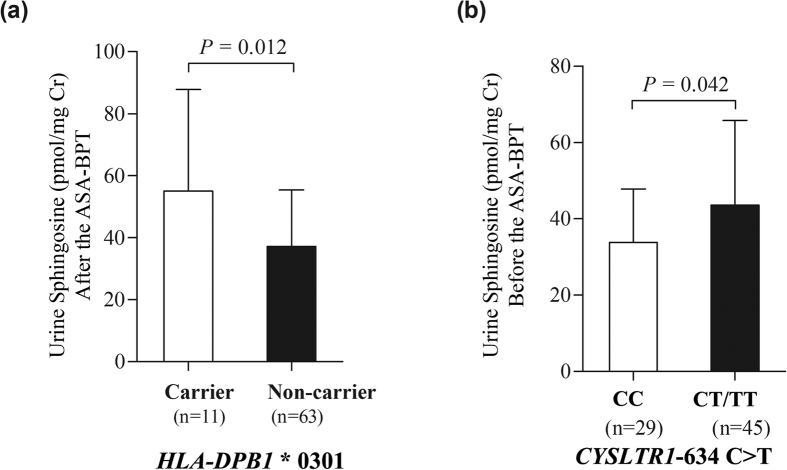
Association of urine sphingosine level (**a**) at the basal status with HLA-DBP1*0301 and (**b**) at onset of reaction symptoms after the ASA-BPT test with *CYSLTR1*-634C > T polymorphism.

We examined the association of S1P and sphingosine with HLA-DBP1*0301 and CYSLTR1-634C > T polymorphism in 89 asthmatic patients comprising of 44 patients with AERD and 45 with ATA (Table E3). In terms of HLA-DBP1*0301, patients with HLA-DBP*0301 alleles showed increased urine sphingosine levels at onset of reaction symptoms following the ASA-BPT compared to non-carriers (*P* = 0.012, Fig. 5a). In terms of *CYSLTR1* −634C > T, patients with either heterozygous CT or homozygous TT genotypes demonstrated higher levels of urine sphingosine at the basal status before the ASA-BPT than those with the homozygous CC genotype (*P* = 0.042, Fig. 5b).

## Discussion

AERD is well known as a distinct phenotype and endotype of asthma; it is characterized by eosinophilic inflammation, enhanced leukotriene production and activation of airway mast cells^22^,^23^. Recently, novel mechanisms have been proposed, such as systemic inflammation, aberrant functions of platelets and overexpressed platelet activation^24^,^25^,^26^. Aspirin challenge, for instance the ASA-BPT, is the gold standard for confirming aspirin hypersensitivity, however, there are still risks for bronchospasm as well as various contraindications^27^. In addition, no *in vitro* test has been routinely used for aspirin hypersensitivity despite the fact that AERD patients usually develop severe asthma and a higher frequency of emergency visits, hospitalizations and corticosteroid bursts^15^,^16^,^17^. Thus, non-invasive, serological tests should be investigated to determine an ideal biomarker for the AERD phenotype. In this study, we demonstrated the association of SL metabolites in AERD pathogenesis, especially serum S1P and urine sphingosine, could be potential biomarkers for AERD phenotype.

In the present study, we observed the metabolic disturbance of SL in AERD patients. By comparing changes in the SL metabolites following the ASA-BPT, we found a distorted pattern of ceramides and SMs after the ASA-BPT, with a decreased pattern in the AERD group (Fig. E1). In a pairwise comparison before and after the ASA-BPT in the AERD patients, the levels of serum SM (d18:0/18:0, d18:0/18:1, d18:0/24:0, d18:0/24:1) were significantly decreased following the ASA-BPT (Table 2, Fig. 1a). Furthermore, serum SM levels showed positive correlations with the concentration of methacholine needed to produce a 20% fall in FEV_1_ from baseline, PC_20_. This indicates that decreased SM levels in AERD patients associated with enhanced airway hyperreactivity. There are several reports supporting the relationship between SL and airway hyperreactivity. Worgall *et al*. demonstrated the association of impaired SL synthesis with airway hyperreacitivity by using *de novo* SPT inhibitor and an SPT knockout mouse model^28^. They found that impaired SPT activity induced decreased serum ceramides and sphingosine levels, finally resulting in bronchial hyperreactivity. The direct effect of S1P on airway hyperreactivity has been also reported; S1P administration increased airway reactivity in a dose – and time-dependent manner in mouse lungs, in which IL-17 may be involved^10^. In the present study, we firstly demonstrated the correlation of serum SM levels with methacholine-induced airway hyperreactivity.

The present study demonstrated the potential of serum S1P and urine sphingosine as biomarkers for identifying AERD. Enhanced levels of serum S1P, sphingosine and urine sphingosine were found in patients with AERD compared with those in ATA patients (Table 2, Fig. 2a). We also measured the serum S1P level in 29 healthy controls to validate the data and found a significantly increased level of serum S1P in AERD compared to healthy control groups (Fig. E2). In addition, the serum S1P and urine sphingosine levels were significantly correlated with a greater decrease in FEV_1_ (%) values after the ASA-BPT (Fig. 2b). Because the ASA-BPT has been validated and widely used in AERD diagnosis, these results indicate that the serum S1P and urine sphingosine levels are reliable, and safer markers to diagnose the AERD phenotype, without the fear of adverse reactions. We then confirmed the potential of serum S1P and urine sphingosine to differentiate AERD from ATA using ROC analysis, revealing that urine sphingosine is more optimal, with a relatively high sensitivity and specificity (Fig. 3).

Pezato *et al*.^24^ demonstrated the underlying systemic inflammation of AERD patients. AERD patients showed a differential baseline inflammatory pattern such as an enhanced baseline level of urine leukotriene E4^29^. Similarly, the increased levels of S1P and sphingosine in the AERD patients showed positive correlations with the serum 15-HETE level, while the levels of serum SMs were negatively correlated with the serum 15-HETE level (Table 3). These findings demonstrate that alteration of SL metabolism (shifting SM into sphingosine and S1P) may be involved in the underlying mechanism of AERD. The SL metabolic pathway is an important cellular pathway that represents a highly coordinated system in which S1P induces cell survival, while ceramide induces programmed cell death^2^. According to previous knowledge of SL metabolism, the interconversion of SM into ceramide and S1P is under the control of anabolic and catabolic pathways, in which many kinases and phosphatases are involved, finally determining the cellular value of S1P. Together, S1P and sphingosine may participate in the systemic inflammatory response of AERD.

Next, we investigated the involvement of SLs in various aspects of AERD pathogenesis. Periostin has been suggested as a biomarker for disease severity in asthma as well as the best predictor of airway eosinophilia in asthmatics^30^. Periostin is necessary for house-dust mite-induced airway hyperresponsiveness in mice through dendritic cell activation^31^. A recent finding has demonstrated that serum periostin could also be used as a biomarker for the AERD phenotype and disease severity^21^. In our study, the serum S1P and urine sphingosine levels were positively correlated with the serum periostin level (Fig. 2c). Although the mechanisms of periostin in AERD are not fully understood, it is proposed that exposure to Th2-driven inflammation enhances the overexpression of the *POSTN* gene, resulting an increased level of periostin^21^. In addition, IL-13 may induce release of periostin from bronchial epithelial cells^32^. S1P is known to be associated with airway hyperactivity and Th2 cytokines in mouse lung^10^,^11^,^33^. S1P induction could lead to the release of Th2 cytokines, such as IL-4 and IL-13^10^,^34^. Therefore, we speculate that a high level of serum S1P in AERD could generate a Th2 cytokines-rich milieu for the production of periostin, subsequently contributing to more severe airway inflammatory responses.

The suggested genetic mechanisms of AERD are the polymorphisms of genes coding for CYSLTRs and the HLA system. HLA-DBP1*0301 has been proposed as an independent risk factor for the AERD phenotype^19^. The frequency of HLA-DPB1*0301 was more prominent in AERD patients than in ATA patients^19^. The AERD patients who have HLA-DBP1*0301 showed a higher requirement for leukotriene receptor antagonists for the management^35^. In addition, the polymorphism of *CYSLTR1* at position -634 (*C* > *T*) is associated with the AERD phenotype^20^. The frequency of minor alleles of this SNP was higher in male AERD patients than in male ATA patients. Female patients with the *CYSLTR1* -634*CT or TT* genotypes showed an increased total IgE level, perhaps resulting from the elevated production of the Th2 cytokine in this group^20^. Similarly, our study revealed that the urine sphingosine levels were increased in *HLA*-DPB1*0301 carriers and *CYSLTR1* -634 *(C* > *T)* minor carriers (Fig. 5). The significance seems to be marginal due to limited number of study subjects, thus, further study in a large study cohort is needed to clarify the mechanistic basis regarding how the *HLA*-DPB1*0301 and *CYSLTR1* gene polymorphisms can influence the level of the urine sphingosine.

Platelets contribute to the pathogenesis of AERD through various pathways, including the formation of platelet-leukocyte aggregates, abnormal function of platelets *in vitro* and overexpression of platelet activation^25^,^26^,^36^. After platelet activation, intracellular S1P is released into the environment^37^,^38^. *In vitro* studies have found that arachidonic acid could stimulate platelets to secrete S1P through the activation of the thromboxane receptor^38^. Recently, integrative pathway-based and conditional Gaussian Bayesian network analyses using multi-omics data have indicated that an altered SL metabolism represents an underlying feature of uncontrolled asthma and a therapeutic response to albuterol^39^. Interestingly, the S1P pathway was significantly enriched in the prostaglandin E2 expression signature; thirteen up-regulated and eight down-regulated genes in response to PGE2 treatment were determined to be part of the S1P pathway^39^. These findings may suggest a cross-talk between SL and eicosanoid metabolism in the pathogenesis of AERD in which platelet activation and subsequent platelet-leukocyte aggregates might be involved; however, further studies are required to elucidate the relationship between circulating S1P and the underlying systemic inflammation of AERD patients, as well as the downstream effects of S1P in AERD pathogenesis. Understanding the nature of SL metabolism and its pathological effect on the AERD phenotype would be useful in developing novel therapeutic strategies as well as early diagnostic tools.

In conclusion, to our knowledge, this study is the first to demonstrate that S1P and sphingosine are enhanced in AERD patients. SLs may play important roles in AERD pathogenesis. Serum S1P and urine sphingosine may be potential biomarkers for the AERD phenotype.

## Materials and Methods

### Study subjects and sample collection

We recruited 45 AERD and 45 ATA patients from the Ajou University Medical Center (Suwon, Korea) to investigate their SL metabolite profiles. All patients were evaluated for clinical history, chest radiography and pulmonary function tests. Subjects with stable asthma and a baseline FEV_1_ ≥ 50% predicted values underwent methacholine challenge in accordance with American Thoracic Society standards^40^. Subjects inhaled aerosolized normal saline, followed by aerosolized saline containing methacholine in doubling doses from 0.03 to 25.0 mg/mL. Methacholine was administered until either FEV_1_ fell by 20% of baseline or until the final dose was administered. If a subject experienced a drop in FEV_1_ from baseline of ≥ 20% before or immediately after the administration of the final dose, the test was considered positive^40^. For the ASA-BPT test, the patients were avoided the use of all anti-asthmatic drugs, including leukotriene modifiers, for at least 3 days before. The ASA-BPT was performed with increasing doses of ASA solution (up to 300 mg/mL) using previously described methods, and the tests were defined as positive when FEV_1_% decreased by more than 15% after the challenge^41^. The serum and urine samples were collected from the patients at different times; their basal statuses were examined before the ASA-BPT, and their onset of symptoms as well as FEV_1_ were examined after the ASA-BPT. For the ATA patients, the second sampling was performed 4 hours after the last dose of lysine-aspirin. We also recruited 29 normal healthy controls to validate the significant increase of serum S1P at the basal status before the ASA-BPT test (Supplementary Materials and Methods).

Serum total IgE was measured using the ImmunoCAP system (ThermoFisher Scientific, Uppsala, Sweden), and a range of 2–5000 kU/L. All of the subjects provided written informed consent and the study protocol was approved by the Institutional Review Board of Ajou University Hospital (AJIRB-GEN-SMP-13–108), and all methods were performed in accordance with the relevant guidelines and regulations.

### Quantification of SL metabolites

S1P and sphingosine in both serum and urine samples were quantified using the Mass Hunter Quantitative Analysis B.07.00 system (Agilent Technologies, Santa Clara, CA, USA). D-erythro-Sphingosine C-17 was used as the internal standard for sphingosine and S1P (Cayman Chemical., Ann Arbor, MI, USA). We also used Mass Hunter Quantitative Analysis B.07.00 to quantify the 15-HETE in which the deuterated internal standard 15-HETE-d8 (Cayman Chemical) was used. For the urine samples, creatinine was also quantified to normalize to the actual concentrations of each metabolite and 1,3-dimethyl-2-imidazolidinone (Sigma-Aldrich, St. Louis, MO, USA) was used as the internal standard for creatinine. The concentration of each metabolite was determined from calibration curves using linear regression analysis.

Ceramide and SMs were quantified through a liquid chromatography-tandem mass spectrometry (LC-MS/MS) system equipped with 1290 HPLC (Agilent, Waldbronn, Germany) and QTRAP 5500 (AB Sciex, Toronto, Canada). Multiple reaction monitoring (MRM) was performed in the positive ion mode and the extracted ion chromatogram corresponding to the specific transition for each lipid was used for quantification. The calibration range for each lipid was 0.1–1,000 nM (*r* ≥ 0.99). Data analysis was performed using either Analyst 1.5.2 or Xcalibur software.

The limit of detection was determined as the lowest concentration that can still be detected and distinguished with the limit of blank.

Additional details of the methods used are available in the Methods section of this article’s online supplementary information.

### Measurement of serum periostin levels

Serum periostin levels were measured with a proprietary sandwich ELISA (Shino-test, Kanagawa, Japan), as previously described^21^. ELISA was performed in duplicate. The reported limiting level of periostin for detection using this ELISA system is 100 pg/mL^42^.

### Genotyping

Blood samples were collected from 89 asthmatic patients comprising AERD and ATA before the ASA-BPT test. Genomic DNA was isolated from the peripheral blood using the Puregene DNA purification kit (Gentra, Minneapolis, MN, USA), according to the manufacturer’s instructions.

High-resolution typing for HLA-DBP1*0301 was performed as previously described, using the amplified gene product of locus specific primers and direct sequencing with the ABI 3100 Genetic Analyzer (Applied Biosystems, Foster City, CA, USA)^19^. Genotyping of *CYSLTR1* -634C > T gene polymorphism (rs321029) was performed as previously described, using a primer extension method with a SNaPshot ddNTP primer extension kit (Applied Biosystems)^20^.

### Statistical analysis

We log-transformed the data of methacholine concentration needed to produce a 20% fall in FEV_1_ and the serum total IgE level to correct their skewed distributions. Student’s t-test was used to compare continuous outcomes across the levels of each metabolite. General linear regression analysis was performed to adjust for confounding factors, sex and age, to compare the metabolite levels between the AERD and ATA patients. Paired t- test was used to compare paired continuous variables. Spearman’s rho correlation analysis identified the associations between continuous variables. To evaluate the diagnostic value of serum S1P and urine sphingosine for identifying AERD, a ROC analysis was used. All *P* values < 0.05 were considered to be statistically significant. Statistical analyses were performed using SPSS software version 20.0 (IBM Corp., Armonk, NY, USA). GraphPad Prism 5.0 software (GraphPad Inc., San Diego, CA, USA) was used to produce graphs.

## Additional Information

**How to cite this article**: Trinh, H. K. T. *et al*. Exploration of the Sphingolipid Metabolite, Sphingosine-1-phosphate and Sphingosine, as Novel Biomarkers for Aspirin-exacerbated Respiratory Disease. *Sci. Rep*. **6**, 36599; doi: 10.1038/srep36599 (2016).

**Publisher’s note:** Springer Nature remains neutral with regard to jurisdictional claims in published maps and institutional affiliations.

## Supplementary Material

Supplementary Information

## Figures and Tables

**Table 1:** Clinical demographics of the study subjects.

	AERD	ATA	*P* value
	(N = 45)	(N = 45)	AERD *vs* ATA
Age (year)	40.27 ± 13.43 (16–69)	46.07 ± 18 (18–74)	**0.045**
Sex (Female)	28 (62.2%)	32 (71.1%)	0.371
Atopy	26 (57.8%)	17 (38.6%)	0.071
Total IgE (KU/L)*	2.31 ± 0.57	2.06 ± 0.65	0.458
Baseline FEV_1_ (% Pred)	84.65 ± 17.94	85.84 ± 16.55	0.79
% Fall (FEV_1_)§	22.57 ± 10.19	4.67 ± 3.56	**<0.001**
PC_20_ (mg/mL)*	0.25 ± 0.67	0.69 ± 0.55	**0.004**
Rhinosinusitis	39/45 (86.7%)	26/42 (61.9%)	**0.013**
Nasal polyposis	16/45 (37.8%)	7/42 (16.7%)	**0.033**

AERD, aspirin-exacerbated respiratory disease; ATA, aspirin- tolerant asthma; NA, not applicable. The continuous data are presented as the means ± SD. The dichotomous data are presented as numbers (%). The data were analyzed with the t test and Pearson’s chi-squared test.

^§^The percent of FEV_1_ decreased during the ASA-BPT. Values in bold indicate significant *P* value.

^*^Log-transformed data were presented and analyzed with the t test.

**Table 2:** Sphingolipid composition of patients with AERD and ATA before and after the ASA-BPT.

Type of sample	SL metabolites	Before		*P* value† AERD vs ATA	After		*P* value† AERD *vs* ATA	AERD	ATA
		AERD (N = 45)	ATA (N = 45)		AERD (N = 45)	ATA (N = 45)		*P* value‡ before *vs* after	*P* value‡ before *vs* after
Serum (ng/mL)	S1P	101.86 ± 23.48	85.69 ± 20.76	0.001	112.62 ± 20.50	95.71 ± 16.64	<**0.001**	0.022	**0.026**
	Sphingosine	4.23 ± 6.66	1.47 ± 1.13	0.009	3.24 ± 3.53	1.58 ± 1.21	**0.004**	0.053	0.286
	C16:0 Ceramide	56.48 ± 47.92	46.02 ± 40.02	0.391	41.72 ± 35.71	39.39 ± 31.36	0.773	0.129	0.347
	C18:0 Ceramide	596.9 ± 426.14	561.39 ± 458.85	0.753	459.31 ± 471.8	698.98 ± 690.07	0.091	0.189	0.258
	C18:1 Ceramide	26.16 ± 23.32	17.89 ± 14.33	0.096	19.7 ± 16.23	21.45 ± 14.5	0.600	0.182	0.284
	C20:0 Ceramide	431.63 ± 289.19	425.25 ± 334.92	0.856	401.64 ± 437.73	528.77 ± 451.32	0.323	0.725	0.236
	C24:0 Ceramide	43487.36 ± 68722.91	46939.51 ± 65163.38	0.872	28827.32 ± 31166.87	45757.38 ± 46134.42	0.111	0.135	0.905
	C24:1 Ceramide	16428.46 ± 22695.26	14179.54 ± 19020.02	0.469	13063.48 ± 17229.03	15799.6 ± 15430.6	0.565	0.225	0.443
	SM (d18:0/16:0)	634689.34 ± 834083.29	574655.49 ± 496300.08	0.478	418167.27 ± 373508.9	589256.12 ± 543663.91	0.243	0.050	0.887
	SM (d18:0/18:0)	117147.07 ± 100604.79	119072.53 ± 112764.54	0.758	87509.14 ± 57956.94	118776.79 ± 93683.63	0.235	**0.035**	0.990
	SM (d18:0/18:1)	288429.15 ± 334236.26	251856.35 ± 230136.98	0.346	196502.31 ± 162071.74	254202.24 ± 186918.08	0.348	**0.038**	0.962
	SM (d18:0/24:0)	229334.6 ± 239649.44	192279.45 ± 160687.77	0.237	156231.31 ± 129139.24	199692.37 ± 163019.24	0.417	**0.028**	0.813
	SM (d18:0/24:1)	401790.38 ± 393446.33	300081.46 ± 261921.51	0.170	266693.65 ± 207376.38	318932.87 ± 278792.52	0.469	**0.013**	0.745
	Lyso SM	1348.72 ± 1637.08	1040.93 ± 849.71	0.239	908.74 ± 716.62	1135.74 ± 955.2	0.341	0.060	0.606
Urine*	S1P	34.19 ± 9.86	34.66 ± 12.43	0.629	41.19 ± 1.19 1	40.32 ± 0.32 6	0.726	**0.001**	**0.005**
(pmol/mg Cr)	Sphingosine	48.72 ± 21.58	31.29 ± 13.66	<**0.001**	50.11 ± 0.11 8	30.16 ± 0.16 1	<**0.001**	0.532	0.264
	C14:0 Ceramide	154.67 ± 54.67 1	166.96 ± 66.96 9	0.596	144.98 ± 44.98	242.02 ± 42.02 6	0.355	0.424	0.684
	C16:0 Ceramide	2807.85 ± 3699.32	3304.29 ± 5801.60	0.446	2932.36 ± 3180.57	5292.69 ± 9283.27	0.342	0.335	0.234
	C18:0 Ceramide	399.31 ± 38.46	471.51 ± 86.96	0.524	420.10 ± 95.74	839.06 ± 517.83	0.274	0.437	0.151
	C20:0 Ceramide	650.46 ± 16.87	601.17 ± 305.52	0.725	726.95 ± 49.77	916.92 ± 80.26	0.534	0.275	0.178
	C24:0 Ceramide	1861.21 ± 1965.82	2214.40 ± 3925.29	0.340	1962.82 ± 1545.23	3864.77 ± 7114.12	0.193	0.281	0.137
	C24:1 Ceramide	343.32 ± 95.64	726.78 ± 381.57	0.058	378.00 ± 60.49	1215.88 ± 3931.01	0.250	0.239	0.314
	Sphinganine	1184.94 ± 03.38	464.92 ± 0.14	0.072	1255.57 ± 2057.80	725.73 ± 1036.23	0.214	0.513	0.049
	SM (d18:0/16:0)	102343.59 ± 64388.97	97934.29 ± 92854.80	0.662	105220.51 ± 58546.58	111780.95 ± 85951.83	0.593	0.457	0.144
	SM (d18:0/18:0)	24703.85 ± 24562.57	25473.65 ± 32979.63	0.259	23060.26 ± 16354.33	35243.73 ± 47199.35	0.216	0.900	0.143
	SM (d18:0/18:1)	1672.54 ± 1431.48	2409.25 ± 4176.42	0.221	1634.46 ± 1088.91	4708.95 ± 14959.42	0.248	0.673	0.248
	SM (d18:0/24:0)	82282.05 ± 65291.75	75552.38 ± 69356.22	0.719	79694.87 ± 43651.90	96132.00 ± 80454.76	0.351	0.661	0.074
	SM (d18:0/24:1)	40989.74 ± 38049.69	40254.86 ± 46033.41	0.450	38362.31 ± 24894.45	53031.24 ± 66543.38	0.224	0.863	0.115

AERD, aspirin-exacerbated respiratory disease; ATA, aspirin- tolerant asthma;, SM, sphingomyelin; Cr, creatinine.

^*^C14 Ceramide and sphinganine were not detected in human serum samples.

^†^*P* value was calculated by GLM, controlling for sex and age. ^¶^Values in bold indicate significant *P* value.

^‡^*P* value was calculated using a paired t-test.

**Table 3:** Association of baseline levels of serum SL metabolites with clinical features and biomarkers.

	serum S1P†	Sphingosine†	urine S1P†	Urine Sphingosine†	SM (d18:0/16:0)‡	SM (d18:0/18:0)‡	SM (d18:0/18:1)‡	SM (d18:0/24:0)‡	SM (d18:0/24:1)‡	Lyso SM‡
PC _20_ (mg/mL)*	−0.214 (0.075)	−0.230(0.55)	−0.111 (0.406)	−0.211 (0.112)	0.251 (0.051)	0.236 (0.067)	0.273* (0.033)	0.314* (0.014)	0.233 (0.071)	0.252 (0.050)
Baseline FEV_1_ (% Pred)	−0.033 (0.794)	−0.029 (0.818)	−0.048 (0.732)	−0.103 (0.460)	−0.114 (0.422)	−0.020 (0.890)	−0.033 (0.818)	0.023 (0.869)	0.095 (0.505)	−0.062 (0.663)
% Fall (FEV1)	0.451*** (<0.001)	0.247 (0.053)	0.180 (0.207)	0.553*** (<0.001)	−0.205 (0.152)	−0.199 (0.166)	−0.191 (0.183)	−0.120 (0.406)	−0.053 (0.716)	−0.182 (0.205)
Serum 15-HETE (ng/ml)	0.152 (0.153)	0.459*** (<0.001)	−0.124 (0.291)	0.006 (0.957)	−0.338** (0.005)	−0.299* (0.013)	−0.263* (0.030)	−0.197 (0.108)	0.001 (0.996)	−0.282* (0.020)
Serum periostin (ng/ml)	0.233* (0.033)	0.237* (0.030)	−0.102 (0.406)	0.405** (0.001)	−0.146 (0.247)	−0.115 (0.361)	−0.121 (0.338)	−0.102 (0.417)	−0.051 (0.688)	−0.101 (0.422)

^†^The data presented as Spearman’s rho correlation coefficient *r* (*P* value).

^‡^The data presented as Pearson correlation coefficient *r* (*P* value).
