# Supplementary material for: Exploration of the Sphingolipid Metabolite, Sphingosine-1-phosphate and Sphingosine, as Novel Biomarkers for Aspirin-exacerbated Respiratory Disease
Source: Sci Rep. 2016 Nov 10;6:36599. doi: 10.1038/srep36599 (PMC5103193; doi:10.1038/srep36599)
Supplement: Supplementary Information [file srep36599-s1.doc]

**Exploration of the Sphingolipid Metabolite, Sphingosine-1-phosphate and Sphingosine, as Novel Biomarkers for Aspirin-exacerbated Respiratory Disease**

Hoang Kim Tu Trinh ^1*^, Su-Chin Kim ^2*^, Kumsun Cho,^3^ Su- Jung Kim^4^ , Ga-Young Ban,^1^ Hyun- Ju Yoo^4^ , Joo-Youn Cho^3^ , Hae-Sim Park^1†^ , Seung-Hyun Kim ^1,2†^

^1^Department of Allergy and Clinical Immunology, Ajou University School of Medicine, Suwon, Korea; ^2^ Translational Research Laboratory for Inflammatory Disease, Clinical Trial Center, Ajou University Medical Center, Suwon, South Korea;

^3^Department of Pharmacology and Therapeutics, Seoul National University College of Medicine and Hospital, Seoul, Korea; ^4^ Asan Institute for Life Sciences, Asan Medical Center, University of Ulsan College of Medicine, Seoul, Korea.

*Both authors contributed equally to this work.

†: Corresponding authors:

**Supplementary methods**

*Quantification of sphingosine-1-phosphate (S1P) and sphingosine using Q-TOF MS*

The serum and urine samples were extracted and diluted five times with 80% methanol and water, respectively. All of the samples were mixed for 10 min and centrifuged at 18,341 × *g* for 20 min at 4°C.

Each sample (5 μL) was injected into the column held at 40°C and was eluted with formic acid (0.1%) and ammonium formate (20 mM) in water (solvent A), and formic acid (0.1%) in methanol (solvent B) over 21 minutes. The metabolites were eluted at a flow rate of 0.4 mL/minute using the following gradient: 2% - 98% B from 0.1 to 13 minutes, and 98% B held constant for 2 minutes followed by a return to 2% B from 15.1 to 17 minutes. Chromatographic separation of the metabolites was executed with an ACQUITY UPLC BEH C18, 2.1x50-mm, 1.7-µm (Waters, Milford, MA, USA) analytical column using the Agilent 1290 Infinity II UHPLC system (Agilent Technologies, Santa Clara, CA, USA). Eluents were transported to an Agilent 6530 quadrupole time-of-flight (Q-TOF) mass spectrometer (MS). The instrument settings have been elucidated in detail in a previous study [^1^](#_ENREF_1).

All of the raw data were converted into the compound exchange file format using Mass Hunter DA reprocessor software B.04.00 (Agilent Technologies). Each metabolite feature (m/z × intensity × time) of the transformed data was retention time (RT) aligned and converted into a matrix of detected peaks versus compound identification using Mass Profiler Professional (MPP) software B.12.01 (Agilent Technologies). All of the data were centered, scaled, and log 2 transformed.

The compounds of the specific metabolites were confirmed by comparing the chromatographism and their mass spectra to those obtained using commercially available reference standards. D-erythro-Sphingosine C-17 was used as the internal standard for sphingosine and S1P (Cayman Chemical Company, Ann Arbor, MI, USA). In urine samples, creatinine was also quantified to normalize to the actual concentrations of each metabolite and 1,3-dimethyl-2-imidazolidinone (Sigma-Aldrich, St. Louis, MO, USA) was used as the internal standard for creatinine. The concentration of each metabolite was decided from calibration curves using linear regression analysis.

*Quantification of sphingomyelin and ceramide using LC-MS/MS*

Human serum or sputum supernatant was homogenized well and internal standard solutions (500 nM of C17 ceramide solution for other sphingolipids) were added to the samples before extraction. Sphingolipids were extracted by the Bligh and Dayer method [^2^](#_ENREF_2). After lipid extraction, organic solutions containing lipids were dried using a vacuum centrifuge and stored at –20°C until LC-MS/MS analysis. The dried matter was reconstituted with methanol and injected into the LC-MS/MS system. All of the lipid standards including the internal standards were purchased from Avanti-Polar Lipids and Sigma-Aldrich.

The lipid levels were determined using an LC-MS/MS system equipped with a 1290 HPLC (Agilent, Waldbronn, Germany) and QTRAP 5500 (AB Sciex, Toronto, Canada). A reverse-phase column (Pursuit5 C18, 150 × 2.1 mm) was used with mobile phase A (5 mM ammonium formate/methanol/tetrahydrofuran (500/200/300) and mobile phase B (5 mM ammonium formate/methanol/ tetrahydrofuran (100/200/700). The LC was run at 200 µL/min and 35°C. The LC gradient was as follows: 50 % of A for 0 min, 50 % of A for 5 min, 50 to 30 % of A for 3 min, 30 % of A for 7 min, 30 to 10 % of A for 7 min, 10 % of A for 3 min, 10 to 50 % of A for 0.1 min), and 50 % of A for 4.9 min. Multiple reaction monitoring (MRM) was performed in the positive ion mode and the extracted ion chromatogram corresponding to the specific transition for each lipid was used for quantification. The calibration range for each lipid was 0.1-1,000 nM (r^2^ ≥ 0.99). Data analysis was performed by either Analyst 1.5.2 or Xcaliber software.

*Star and symbol plotting*

To determine how SL metabolite levels changed during the ASA-BPT, we used star and symbol plotting considering the induction fold of the SL metabolite level after the ASA-BPT for the AERD and ATA groups. The far ends of the lines were joined together to produce the star and symbol patterns using the program Microsoft Excel (Microsoft Corp., Redmond, WA, USA).

**Reference**

1 Cho, K. *et al.* Antihyperglycemic mechanism of metformin occurs via the AMPK/LXRalpha/POMC pathway. *Sci Rep.* **5**, 8145.(2015).

2 Bligh, E. G. & Dyer, W. J. A rapid method of total lipid extraction and purification. *Can J Biochem Physiol.* **37**, 911-917.(1959).

**Supplementary figure legends**

**Fig. E1.** Star and symbol plot of the AERD and ATA groups based on the induction folds of the SL metabolites during the ASA-BPT. a) Serum SL metabolites and b) urine SL metabolites. The continuous line represents the changes in the SL metabolites in patients with AERD and the dashed line represents those of patients with ATA. If the ratio is equal to 1, the levels of SL metabolites did not differ before and after the Lys-ASA-BPT. If the ratio is higher or lower than 1, the levels of SL metabolites were decreased or increased after the Lys-ASA-BPT, respectively. In AERD, we found a distorted pattern of ceramides and SM after the ASA-BPT. The SL metabolites are numbered as below: 1. Sphingosine-1-phosphate (S1P); 2: Sphingosine; 3: C16:0 Ceramide; 4: C18:0 Ceramide; 5: C18:1 Ceramide; 6: C20:0 Ceramide; 7: C24:0 Ceramide; 8: C24:1 Ceramide; 9: SM (d18:0/16:0); 10: SM (d18:0/18:0); 11: SM (d18:0/18:1), 12: SM (d18:0/24:0); 13: SM (d18:0/24:1), 14: Lyso SM.

**Fig. E2.** Serum levels of sphingosine-1-phosphate from AERD, ATA and NC at the basal status before the ASA-BPT.

**Supplementary tables**

**Table E1. Spearman's rho correlation coefficients between serum SL metabolites levels at the basal status before the ASA-BPT.**

**Table E2. Spearman's rho correlation coefficients between urine SL metabolites levels at the basal status before the ASA-BPT.**

**Table E3. Genetic association of S1P and sphingosine with HLA-DPB1*0301 and CYSTLR1-634C>T polymorphism**

|  | | | | | | | |  |  |  |  |  |  |  |
| --- | --- | --- | --- | --- | --- | --- | --- | --- | --- | --- | --- | --- | --- | --- |
| **SL metabolites** | S1P | Sphingosine | C16 Ceramide | C18 Ceramide | C18:1 Ceramide | C20 Ceramide | C24 Ceramide | C24:1 Ceramide | SM(d18:0/16:0) | SM(d18:0/18:0) | SM(d18:0/18:1) | SM(d18:0/24:0) | SM(d18:0/24:1) | Lyso SM |
| S1P | 1.000 (-) |  |  |  |  |  |  |  |  |  |  |  |  |  |
| Sphingosine | .144 (.175) | 1.000 (-) |  |  |  |  |  |  |  |  |  |  |  |  |
| C16 Ceramide | -.157 (.201) | -.108 (.381) | 1.000 (-) |  |  |  |  |  |  |  |  |  |  |  |
| C18 Ceramide | -.160 (.193) | .115 (.351) | .387** (.001) | 1.000 (-) |  |  |  |  |  |  |  |  |  |  |
| C18:1 Ceramide | -.102 (.410) | -.095 (.442) | .531** (.000) | .492** (.000) | 1.000 (-) |  |  |  |  |  |  |  |  |  |
| C20 Ceramide | -.122 (.320) | -.084 (.495) | .346** (.004) | .556** (.000) | .586** (.000) | 1.000 (-) |  |  |  |  |  |  |  |  |
| C24 Ceramide | .037 (.767) | -.202 (.099) | .108 (.379) | .186 (.130) | .253* (.037) | .436** (.000) | 1.000 (-) |  |  |  |  |  |  |  |
| C24:1 Ceramide | .055 (.655) | -.186 (.129) | .370** (.002) | .238 (.050) | .299* (.013) | .337** (.005) | .821** (.000) | 1.000 (-) |  |  |  |  |  |  |
| SM(d18:0/16:0) | -.004 (.971) | -.374** (.002) | .331** (.006) | .097 (.429) | .447** (.000) | .432** (.000) | .560** (.000) | .422** (.000) | 1.000 (-) |  |  |  |  |  |
| SM(d18:0/18:0) | -.022 (.856) | -.311** (.010) | .530** (.000) | .353** (.003) | .539** (.000) | .514** (.000) | .535** (.000) | .453** (.000) | .862** (.000) | 1.000 (-) |  |  |  |  |
| SM(d18:0/18:1) | .035 (.775) | -.332** (.006) | .335** (.005) | .297* (.014) | .504** (.000) | .536** (.000) | .651** (.000) | .488** (.000) | .914** (.000) | .908** (.000) | 1.000 (-) |  |  |  |
| SM(d18:0/24:0) | -.078 (.526) | -.241* (.048) | .530** (.000) | .358** (.003) | .450** (.000) | .420** (.000) | .613** (.000) | .632** (.000) | .790** (.000) | .871** (.000) | .824** (.000) | 1.000 (-) |  |  |
| SM(d18:0/24:1) | -.088 (.475) | -.201 (.100) | .576** (.000) | .489** (.000) | .481** (.000) | .430** (.000) | .447** (.000) | .564** (.000) | .581** (.000) | .760** (.000) | .683** (.000) | .909** (.000) | 1.000 (-) |  |
| Lyso SM | .030 (.807) | -.328** (.006) | .370** (.002) | .038 (.760) | .474** (.000) | .405** (.001) | .595** (.000) | .446** (.000) | .925** (.000) | .786** (.000) | .820** (.000) | .706** (.000) | .476** (.000) | 1.000 (-) |
| The data presented as Spearman's rho correlation coefficient γ^2^ (*P* value).  * Values of all significant correlations are given with degree of significance indicated. (* *P* < 0.05, ** *P* < 0.01, *** *P* < 0.001). | | | | | | | | | | | |  |  |  |

**Table E1. Spearman's rho correlation coefficients between serum SL metabolites levels at the basal status before the ASA-BPT.**

**Table E2. Spearman's rho correlation coefficients between urine SL metabolites levels at the basal status before ASA-BPT.**

|  | | | | |  |  |  |  |  |  |  |  |  |  |
| --- | --- | --- | --- | --- | --- | --- | --- | --- | --- | --- | --- | --- | --- | --- |
| **SL metabolites** | S1P | Sphingosine | C14 Ceramide | C16 Ceramide | C18 Ceramide | C20 Ceramide | C24 Ceramide | C24:1 Ceramide | Sphinganine | SM(d18:0/16:0) | SM(d18:0/18:0) | SM(d18:0/18:1) | SM(d18:0/24:0) | SM(d18:0/24:1) |
| S1P | 1.000 (-) |  |  |  |  |  |  |  |  |  |  |  |  |  |
| Sphingosine | .036 (.760) | 1.000 (-) |  |  |  |  |  |  |  |  |  |  |  |  |
| C14 Ceramide | -.075 (.591) | -.113 (.415) | 1.000 (-) |  |  |  |  |  |  |  |  |  |  |  |
| C16 Ceramide | -.052 (.700) | -.064 (.634) | .877** (.000) | 1.000 (-) |  |  |  |  |  |  |  |  |  |  |
| C18 Ceramide | -.037 (.790) | -.028 (.842) | .854** (.000) | .893** (.000) | 1.000 (-) |  |  |  |  |  |  |  |  |  |
| C20 Ceramide | .024 (.862) | .040 (.772) | .870** (.000) | .884** (.000) | .851** (.000) | 1.000 (-) |  |  |  |  |  |  |  |  |
| C24 Ceramide | -.018 (.893) | -.043 (.751) | .840** (.000) | .932** (.000) | .897** (.000) | .881** (.000) | 1.000 (-) |  |  |  |  |  |  |  |
| C24:1 Ceramide | .015 (.913) | -.147 (.274) | .801** (.000) | .885** (.000) | .857** (.000) | .864** (.000) | .893** (.000) | 1.000 (-) |  |  |  |  |  |  |
| Sphinganine | -.095 (.476) | -.106 (.429) | .549** (.000) | .605** (.000) | .605** (.000) | .579** (.000) | .644** (.000) | .548** (.000) | 1.000 (-) |  |  |  |  |  |
| SM(d18:0/16:0) | -.038 (.778) | -.110 (.413) | .800** (.000) | .846** (.000) | .759** (.000) | .845** (.000) | .840** (.000) | .867** (.000) | .707** (.000) | 1.000 (-) |  |  |  |  |
| SM(d18:0/18:0) | .006 (.964) | -.144 (.284) | .822** (.000) | .807** (.000) | .780** (.000) | .870** (.000) | .829** (.000) | .847** (.000) | .636** (.000) | .931** (.000) | 1.000 (-) |  |  |  |
| SM(d18:0/18:1) | .014 (.919) | -.209 (.115) | .784** (.000) | .813** (.000) | .796** (.000) | .833** (.000) | .831** (.000) | .885** (.000) | .613** (.000) | .928** (.000) | .945** (.000) | 1.000 (-) |  |  |
| SM(d18:0/24:0) | -.109 (.415) | -.206 (.121) | .770** (.000) | .769** (.000) | .687** (.000) | .845** (.000) | .783** (.000) | .802** (.000) | .651** (.000) | .933** (.000) | .960** (.000) | .907** (.000) | 1.000 (-) |  |
| SM(d18:0/24:1) | -.053 (.695) | -.192 (.148) | .783** (.000) | .803** (.000) | .733** (.000) | .852** (.000) | .823** (.000) | .871** (.000) | .641** (.000) | .957** (.000) | .968** (.000) | .953** (.000) | .962** (.000) | 1.000 (-) |
|  | | | | | | |  |  |  |  |  |  |  |  |

The data presented as Spearman's rho correlation coefficient γ^2^ (*P* value). Values of all significant correlations are given with degree of significance indicated.(** *P* < 0.01)

**Table E3. Genetic association of S1P and sphingosine with HLA-DPB1*0301 and CysTLR1-634C>T polymorphism**

|  | | | | | |  |  | |  | |  | |
| --- | --- | --- | --- | --- | --- | --- | --- | --- | --- | --- | --- | --- |
|  |  |  | **HLA-DPB1*0301** | |  | **CYSLTR1 -634 C>T** | | | |  | |  |
| **Type of sample** | **ASA-BPT** | **SL metabolites** | **Carrier** | **None** | ***P* value^†^** | **CC** | | **CT + TT** | | ***P* value^†^** | |  |
|  |  |  | **(N=16)** | **(N=73)** |  | **(N=34)** | | **(N=55)** | |  | |  |
| **Serum**  **(ng/ml)** | **Before** | **S1P** | 89.16 ± 22.71 | 94.67 ± 23.83 | 0.471 | 97.04 ± 27.47 | | 91.61 ± 20.87 | | 0.375 | |  |
|  |  | **Sphingosine** | 3.39 ± 4.54 | 2.73 ± 5.09 | 0.682 | 3.45 ± 6.41 | | 2.47 ± 3.87 | | 0.332 | |  |
|  | **After** | **S1P** | 111.29 ± 19.83 | 102.72 ± 20.47 | 0.090 | 102.72 ± 18.48 | | 105.21 ± 21.79 | | 0.431 | |  |
|  |  | **Sphingosine** | 2.84 ± 2.37 | 2.32 ± 2.85 | 0.484 | 2.63 ± 3.44 | | 2.27 ± 2.27 | | 0.581 | |  |
| **Urine**  **(pmol/mg Cr)** | **Before** | **S1P** | 34.00 ±11.49 | 34.51 ± 11.22 | 0.882 | 32.66 ± 9.78 | | 35.58 ± 11.96 | | 0.158 | |  |
|  |  | **Sphingosine** | 50.18 ± 23.70 | 37.95 ± 18.75 | 0.065 | 33.83 ± 13.98 | | 43.60 ± 22.19 | | **0.042** | |  |
|  | **After** | **S1P** | 40.64 ± 15.65 | 40.76 ± 12.02 | 0.963 | 40.79 ± 11.59 | | 40.71 ± 13.19 | | 0.910 | |  |
|  |  | **Sphingosine** | 55.09 ± 32.68 | 37.21 ± 18.19 | **0.012** | 34.86 ± 15.31 | | 43.09 ± 24.52 | | 0.152 | |  |
| **^†^** *P* value was calculated by generalized linear model, controlling for sex and age. Values in bold indicate significant *P* value | | | | | | | | | | | | |
